# Neutrophil extracellular traps in central nervous system disorders: mechanisms, implications, and emerging perspective

**DOI:** 10.3389/fimmu.2025.1602336

**Published:** 2025-05-21

**Authors:** Shan Qiao, Jing Yuan, Shan-chao Zhang, Ying-ying Lu, Peng Zhou, Tao Xin

**Affiliations:** ^1^ Shandong University of Traditional Chinese Medicine, Jinan, Shandong, China; ^2^ Department of Neurology, The First Affiliated Hospital of Shandong First Medical University and Shandong Provincial Qianfoshan Hospital, Jinan, China; ^3^ Department of Neurosurgery, The First Affiliated Hospital of Shandong First Medical University and Shandong Provincial Qianfoshan Hospital, Jinan, China; ^4^ First Clinical Medical College, Shandong University of Traditional Chinese Medicine, Jinan, Shandong, China; ^5^ Medical Science and Technology Innovation Center, Shandong First Medical University and Shandong Academy of Medical Sciences, Jinan, China

**Keywords:** neutrophil extracellular traps, central nervous system disorders, autoimmune encephalitis, immune mechanism, therapeutic target

## Abstract

Neutrophil Extracellular Traps (NETs), as a crucial defense mechanism of neutrophils, have garnered increasing attention in recent years for their roles in central nervous system (CNS) disorders. This review comprehensively summarizes the fundamental characteristics and formation mechanisms of NETs, while highlighting the latest research advances regarding their involvement in various CNS diseases. Specific mechanistic insights are discussed, including how NETs exacerbate ischemic stroke through immunothrombosis, promote blood-brain barrier disruption in multiple sclerosis, and contribute to neuroinflammation in Alzheimer’s disease. The paper systematically explores the potential mechanistic contributions of NETs to disease pathogenesis and progression, as well as their prospects as diagnostic biomarkers and therapeutic targets. Through an in-depth analysis of the multifaceted roles of NETs in CNS pathologies, this review aims to provide novel insights and references for advancing the understanding, clinical diagnosis, and therapeutic management of central nervous system disorders.

## 1 Introduction

Neutrophils are essential cells that protect the body from pathogen invasion. They also serve a vital function in preserving host tissues, reacting quickly to infection barrier breaches, and sterilizing tissues (1, 2). Soon after infection, neutrophils are drawn to the infection site, where they are activated by identifying pathogen-associated molecular patterns (PAMPs) or damage-associated molecular patterns (DAMPs) via certain pattern recognition receptors (PRRs) on their surface (3). Additionally, when neutrophils are stimulated by different pathogenic bacteria, cytokines, and chemicals, they form a fibrous network termed NETs, which contribute to the immune response to pathogen infection (4, 5). NETosis is the term used to describe this special pathogen eradication mechanism.

Different from necrosis, autophagy, and apoptosis, NETosis is a unique type of cell death (6, 7). By means of its trapping function, it successfully protects the host from pathogen infection. NET overexpression or poor clearance, however, may be a factor in the development of immune-related disorders (8–10). A number of disorders, such as systemic lupus erythematosus, rheumatoid arthritis, atherosclerosis, and cancer, have been linked to NETs, according to earlier research (8, 11). Immunothrombosis, disturbance of the blood-brain barrier, and central inflammatory responses are tightly linked to them (4, 12). Researchers worldwide have recently focused their attention on the role of NETs in illnesses of the central nervous system (CNS), making it a major topic of current research. The most recent research developments on NETs in CNS illnesses are thoroughly reviewed in this article, covering their basic properties, methods of creation, distinct functions in different CNS disorders, and potential as therapeutic targets. The goal is to provide a theoretical framework for bettering clinical diagnosis and therapy of CNS disorders while also comprehending their pathophysiology.

## 2 Characteristics and formation mechanisms of NETs

Neutrophil extracellular traps (NETs) are web-like structures released by neutrophils upon stimulation, first discovered and reported by Brinkmann and colleagues in 2004 (13). NETs are primarily made up of circulating free DNA (cfDNA), citrullinated histones, neutrophil proteases, and other antimicrobial proteins. This structure can catch and immobilize pathogens including bacteria, viruses, and fungus, inhibiting their spread and simplifying their removal. NET creation is a complex mechanism that distinguishes itself from apoptosis and necrosis. It causes neutrophil activity, cell membrane rupture, and the release of DNA and granular proteins. The development of NETs, known as NETosis, is a different immune response mechanism that differs from both apoptosis and necrosis, with mitochondrial malfunction, chromatin decondensation, and nuclear and plasma membrane disruption (5, 14, 15).

The formation of NETs occurs through two main pathways: the classical pathway, known as suicidal NETosis, and the non-classical pathway (12, 13). In suicidal NETosis, the activation of particular signaling pathways (e.g., MEK/ERK, AKT) causes cell membrane rupture and NET release, followed by neutrophil death. In contrast, the non-classical pathway involves the vesicular release of NETs without cell death, allowing neutrophils to survive with intact nuclear and plasma membranes while still contributing to inflammatory processes. Both routes may have a role in central nervous system (CNS) illnesses, although the specific processes may differ depending on the kind of disease and the stimuli (16).

The development and clearing of NETs are dynamic equilibrium processes. Under normal conditions, the production of NETs is strictly controlled to ensure that they play an appropriate role in fighting infections and modifying immunological responses (6, 17). However, in pathological settings, this equilibrium can be upset, resulting in excessive NET creation or insufficient clearance, which can contribute to the development of immune-related illnesses (18, 19). Typically, the blood-brain barrier (BBB) prevents neutrophils from entering the central nervous system. Nonetheless, when the CNS is subjected to trauma or disease, the BBB’s integrity is impaired, allowing neutrophils to enter the brain parenchyma and cerebrospinal fluid. Concurrently, neutrophil infiltration and subsequent NET release aggravate CNS damage and disrupt the BBB (20, 21). NETs have been linked to the pathological processes of many neurological illnesses, including stroke, autoimmune encephalitis, Alzheimer’s disease, multiple sclerosis, traumatic brain injury, and brain tumors (16, 18, 19, 22, 23). However, the precise processes via which NETs contribute to the development and progression of these disorders are unknown. This article will provide an outline of the involvement of NETs in CNS disorders.

## 3 The role of neutrophil extracellular traps in central nervous system disorders

### 3.1 NETs and stroke

Stroke is a cerebrovascular disease that can include focal or global brain tissue impairment due to vascular dysfunction brought on by a variety of reasons (24). Hemorrhagic and ischemic stroke are its two primary varieties. Stroke is a serious risk to a patient’s health and life because of its high incidence, disability, recurrence, and fatality rates (25). New data emphasizes how important NETs are to the pathophysiology of stroke. A focused or global disruption of the cerebral blood supply can result from the migration of peripheral thrombi or the creation of local thrombi, which can cause an ischemic stroke. The impacted brain tissue sustains irreparable damage as a result (26, 27).

Ischemic damage activates an immunological response, allowing immune cells to migrate and infiltrate the brain parenchyma. Within 24 hours of ischemia, active neutrophils and the production of NETs have been seen in ischemic brain tissue, according to studies. NETs secrete a variety of cytotoxic proteases, including histones, myeloperoxidase (MPO), and elastase, which can directly cause endothelial cell damage, increase vascular permeability, and disrupt the blood-brain barrier (BBB) (28, 29). In 2019, Kim et al. discovered that high-mobility group box 1 (HMGB1) is implicated in NET-mediated neuronal injury in ischemic stroke. HMGB1 can cause NET formation through the CXCR4 and TLR4 signaling pathways (30). NETs also contribute to the formation of thrombus by providing a scaffold, promoting the coagulation cascade, and participating in the stroke’s pathological process. Demyanets et al. demonstrated that NETs enhance the coagulation cascade by linking vWF and TFPI, which contributes to thrombosis stabilization (31). Coagulation factor XII is activated by the DNA backbone of NETs in the absence of platelets, and along with coagulation factor XI, it supports the coagulation cascade and the generation of thrombin (32). Furthermore, NET-related changes have been seen in the thrombus structure of ischemic stroke patients as well as individuals with other vascular disorders such coronary artery disease and peripheral artery disease. These changes have been found to have a considerable impact on thrombus stability (33). Furthermore, NETs have been linked to changes in cerebral revascularization and vascular remodeling following ischemic stroke (19).

Intracerebral hemorrhage (ICH) is a frequent cerebrovascular condition, and neuroinflammation is thought to play an important role in its pathogenesis. In experimental ICH models, neutrophil infiltration is considerable in both the core and periphery of hematomas (34). Neutrophil extracellular traps (NETs) have also been discovered as significant factors to the pathogenesis of ICH. An experimental ICH rat model revealed that using DNAse 1 to break down NETs enhanced the fibrinolysis of hematomas induced by tPA, reduced brain swelling, lowered cell death, and improved functional outcomes. They found that NETs impaired the action of tPA in breaking down clots in rats with ICH, and targeting NETs could be a new option to improve fibrinolytic therapy after ICH (35). Wang et al. (36) found that NETs could aggravate tissue plasminogen activator (tPA)-induced brain damage in stroke patients by inhibiting cyclic GMP-AMP synthase (cGAS) (36). Furthermore, RNase has been demonstrated to suppress NET formation in a mouse model of subarachnoid hemorrhage, indicating its potential therapeutic efficacy (37).

In summary, NETs play a pivotal role in ischemic brain injury, and targeting NETs may represent a promising therapeutic strategy for stroke. Despite substantial proof from clinical and animal studies clarifying the mechanisms and roles of NETs in stroke, few NET-targeting treatments have been implemented in clinical practice. More research is critically needed to close the gap and promote treatment development.

### 3.2 NETs and central nervous system autoimmune inflammatory diseases

NETs play a pivotal role in autoimmune diseases, particularly in central nervous system (CNS) autoimmune disorders such as multiple sclerosis (MS), autoimmune encephalitis, and neuropsychiatric systemic lupus erythematosus (NPSLE) (19, 38, 39). In these conditions, NETs act as reservoirs for autoantibodies, exacerbating autoimmune responses by promoting the production and deposition of autoantibodies (19). Furthermore, NETs can exacerbate the inflammatory environment by activating the complement system and promoting the production of pro-inflammatory cytokines, ultimately causing brain tissue damage. For example, in multiple sclerosis, NETs have been found to be strongly correlated with disease activity and severity (38, 39). Studies have shown that NET biomarkers in MS patients’ serum are significantly enhanced, and that they correlate favorably with disease relapse and progression. Further research has revealed that NETs can directly damage neurons and glial cells, contributing to disease exacerbation (19). According to studies on SLE murine models, the activation of the BBB by anti-NR2A/B antibodies results in higher expression of endothelial cell adhesion molecules, which aids in the recruitment, rolling, adhesion, and transmigration of neutrophils, ultimately causing more NETosis within the spinal canal. The release of NETs into the spinal canal contributes to neurotoxicity by causing neuronal cell death, which subsequently leads to the neuropsychiatric symptoms of SLE (40, 41).

The role of NETs in autoimmune encephalitis is also gaining increasing attention. In a study investigating the pathogenesis of anti-NMDAR encephalitis, Qiao et al. identified NETosis in the serum of patients with anti-NMDAR encephalitis (42). Neutrophils from the peripheral blood of individuals with anti-NMDAR encephalitis showed a higher propensity to generate NETs than healthy controls. These individuals had elevated levels of TNF-α, IL-6, and IL-8, which were positively correlated with H3Cit levels. This suggests that NETs are important regulators of the immunoinflammatory processes that underlie anti-NMDAR encephalitis. NET research in autoimmune encephalitis is still in its infancy, nevertheless. Even while certain NET mechanisms in this context have been discovered, there are still a lot of unanswered questions. In order to provide new approaches for the treatment of autoimmune encephalitis, future research is required to clarify the precise molecular pathways of NETs in the pathophysiology of autoimmune encephalitis and to pinpoint particular therapeutic targets against NETs.

### 3.3 NETs and neurodegenerative diseases

Recent studies suggest that NETs may play a role in the pathogenesis of neurodegenerative diseases, such as Alzheimer’s disease (AD) and Parkinson’s disease (PD) (16, 43, 44). Although the precise mechanisms remain incompletely understood, it is hypothesized that NETs contribute to pathological processes such as inflammatory responses and oxidative stress. Research has indicated that the deposition of β-amyloid (Aβ), a characteristic clinical feature of Alzheimer’s disease, is linked to the creation of NET (45). Aβ can cause neutrophil activation and the development of NETs (16, 19). NET constituents including DNA and histones have the ability to attach to Aβ and form complexes that worsen oxidative stress and inflammatory reactions. Additionally, NETs may hasten neuronal degeneration by encouraging the release of oxidative stress products and pro-inflammatory cytokines. The study by Leon et al. revealed an elevation in neutrophil levels in AD brains and in the murine APP/PS1 model (46). In mouse models of Alzheimer’s disease, findings suggest that vascular alterations may encourage neutrophil adhesion and NETosis, with MPO from neutrophils possibly leading to oxidative stress in the vascular system (46).

There is growing evidence that NETs may also play a role in the pathophysiology of Parkinson’s disease. Degeneration of dopaminergic neurons in the substantia nigra and the development of Lewy bodies are hallmarks of Parkinson’s disease (43). Researchers noted that in tissue sections from primary amyloidosis patients, amyloid fibrils significantly prompted NETs release, which relied heavily on the NADPH oxidase system (47). NETs may increase oxidative stress and inflammatory reactions, which could lead to neuronal degeneration. The development of PD is believed to be closely tied to inflammation caused by microglia. Previous research has indicated that CADM3 is involved in the adhesion of inflammatory cells to endothelial cells and in microglia-mediated inflammation (48). According to Qiang et al., CADM3 might play a role in the progression of PD via NETs (43). The research further demonstrated that the co-DEGs of GPR78, CADM3, and CACNA1E connect NETs with Parkinson’s disease and established a nomogram model for diagnosing PD based on these genes. An association between NETs and PD exists, and the expression of genes GPR78, CADM3, and CACNA1E might serve as biomarkers for PD related to NETs. Therefore, in neurodegenerative diseases, reducing inflammation and oxidative stress by preventing NET formation or encouraging the removal of preexisting NET structures may slow the course of the disease.

### 3.4 NETs and traumatic brain injury

The formation of immunothrombosis after traumatic brain injury (TBI) is a critical pathophysiological mechanism contributing to poor outcomes (49). Numerous studies have indicated the possible involvement of NETs in TBI pathology, despite the fact that the relationship between NETs and immunothrombosis in TBI remains unclear (7). Unfavorable prognoses are frequently the result of circulatory disruptions, cerebral blood vessel damage, and blood-brain barrier impairment after traumatic brain injury. After traumatic brain injury (TBI), studies show a large rise in NETs in brain tissues, where they are strongly linked to thrombus formation and inflammatory reactions. The release of NETs by neutrophils is linked to poorer outcomes in TBI and stroke by hindering revascularization and vascular remodeling (23). Coagulopathy is a major factor in the deaths and disabilities linked to TBI. The generation of NETs was driven by HMGB1 from activated platelets, contributing to the procoagulant activity observed in TBI. Moreover, coculture experiments demonstrated that NETs compromised the endothelial barrier and led these cells to adopt a procoagulant phenotype (50). Thus, exploring the processes of NETs in TBI may offer new therapeutic approaches to enhance results as well as deeper understanding of the pathophysiology alterations that occur after TBI.

### 3.5 NETs and brain tumors

In brain tumors, the formation and release of neutrophil extracellular traps (NETs) are closely associated with tumor malignancy, invasiveness, and prognosis (7, 19). Research by Zhang et al. showed that platelets from glioma patients were more likely to induce NET formation than those from healthy individuals, contributing to the hypercoagulable state in these patients (51). In another glioma study, it was observed that high-grade glioma caused more neutrophil infiltration and NETs formation than low-grade glioma. Excessive NETs production enhanced the proliferation, migration, and invasion of glioma cells. Moreover, their findings indicate that NETs generated by neutrophils infiltrating tumors facilitate communication between glioma development and the tumor microenvironment by modulating the HMGB1/RAGE/IL-8 pathway (52). Initial evidence suggested that NET-associated proteins like elastase, proteinase-3, and cathepsin G facilitate brain tumor invasion by breaking down extracellular matrix structures (20). Current research has highlighted the role of NETs in brain tumors, but additional studies are required to understand the molecular mechanisms and aid in developing precise targeted treatments.

## 4 Discussion

Recently, the involvement of NETs in disorders of the central nervous system has attracted growing scientific interest. This extensive review methodically assesses and integrates the newest research developments in this area. Despite significant advancements in comprehending the mechanisms and clinical impacts of NETs in neurological disorders, there remain substantial challenges and unanswered questions that require further research (5, 7, 12, 16, 19, 53).

At the outset, further studies should aim to clarify the exact mechanistic roles that NETs play in central nervous system disorders. Essential tasks include: researching the molecular regulatory mechanisms of NETs in autoimmune disorders; identifying their pathogenic roles to CNS infections; and outlining their pathological roles in neurodegenerative diseases. Special attention should be given to understanding the spatiotemporal patterns of NETs formation, their interactions with neurovascular units, and the downstream inflammatory processes throughout different stages of disease.

Secondly, an in-depth analysis of therapeutic targets related to NETs is a critical area of research. Creating drugs that can either prevent the formation of NETs or promote the clearance of existing NETs structures is important, along with systematically evaluating their clinical applications in central nervous system pathology. By targeting these dual pathways of NETs generation and resolution, we may establish novel therapeutic paradigms that address current treatment limitations in neurological disorders. The ultimate objective is to translate these mechanistic insights into effective clinical interventions that improve patient outcomes. Given the critical role of NETs in central nervous system (CNS) diseases, an increasing number of studies are exploring NETs as a potential therapeutic target (8, 19, 20). There may be a way to reduce inflammatory reactions, safeguard brain tissue, and eventually enhance illness outcomes by preventing NET creation or reducing the amount of NET structures that are already present. A number of therapeutic agents that target NETs, including the DNA-degrading enzyme DNase, are currently undergoing clinical studies (54–57). The structure and function of NETs are disrupted when DNase breaks down their DNA component. DNase has shown promise as a treatment for a number of central nervous system disorders in animal experiments, such as multiple sclerosis and Alzheimer’s. However, there are obstacles to overcome in the therapeutic use of DNase, including best practices for administration, dosage selection, and safety. To confirm the effectiveness and safety of DNase and other NET-targeting treatments in CNS disorders, more clinical research is therefore required. Other tactics, such as protease inhibitors, CXCR2 antagonists, and antibiotic therapies, may also indirectly affect NET production in addition to pharmaceutical interventions (7, 58). Targeting the dual processes of NETs formation and resolution might lead to innovative treatment strategies that tackle the limitations of current therapies for neurological disorders.

## 5 Conclusions and future perspectives

In conclusion, NETs are crucial in disorders of the CNS. Regarding the role of NETs in CNS diseases, considerable advancements have been made in recent years. However, numerous challenges and unresolved questions remain. Therefore, a deeper understanding of the specific mechanisms by which NETs contribute to CNS diseases, as well as the exploration of effective therapeutic strategies targeting NETs, is of paramount importance for improving disease outcomes. Moving forward, with continued advancements in research and technology, we anticipate more precise modulation of NETs’ functions, offering novel approaches and methodologies for the treatment of CNS disorders.
